# Inflammatory and angiogenic biomarkers in diabetic retinopathy

**DOI:** 10.11613/BM.2020.030502

**Published:** 2020-08-05

**Authors:** Snježana Kaštelan, Ivana Orešković, Filip Bišćan, Helena Kaštelan, Antonela Gverović Antunica

**Affiliations:** 1 Department of Ophthalmology, Clinical Hospital Dubrava, Zagreb, Croatia; 2 School of Medicine, University of Zagreb, Zagreb, Croatia; 3 Augenzentrum Mühldorf, Überörtliche Gemeinschaftspraxis, Mühldorf am Inn, Germany; 4 Department of Ophthalmology, General Hospital Dubrovnik, Dubrovnik, Croatia

**Keywords:** diabetic retinopathy, biomarkers, inflammation, angiogenesis

## Abstract

Diabetic retinopathy (DR) is one of the most common microvascular complications of diabetes mellitus (DM) and a leading cause of blindness in working-age adults in developed countries. Numerous investigations have recognised inflammation and angiogenesis as important factors in the development of this complication of diabetes. Current methods of DR treatment are predominantly used at advanced stages of the disease and could be associated with serious side effects. Therefore, new diagnostic methods are needed in order to identify the initial stages of DR as well as monitoring the effects of applied therapy. Biochemical biomarkers are molecules found in blood or other biological fluid and tissue that indicate the existence of an abnormal condition or disease. They could be a valuable tool in detecting early stages of DR, identifying patients most susceptible to retinopathy progression and monitoring treatment outcomes. Biomarkers related to DR can be measured in the blood, retina, vitreous, aqueous humour and recently in tears. As the retina represents a small part of total body mass, a circulating biomarker for DR needs to be highly specific. Local biomarkers are more reliable as indicators of the retinal pathology; however, obtaining a sample of aqueous humour, vitreous or retina is an invasive procedure with potential serious complications. As a non-invasive novel method, tear analysis offers a promising direction in further research for DR biomarker detection. The aim of this paper is to review systemic and local inflammatory and angiogenic biomarkers relevant to this sight threatening diabetic complication.

## Introduction

Diabetic retinopathy (DR) is one of the most common microvascular complications of diabetes mellitus (DM) and a leading cause of visual impairment and preventable blindness in working-age adults in developed countries (*1*). It is characterized by the occurrence of microaneurysms, increased vascular permeability, capillary occlusion, fibrous and neovascular proliferation. Diabetic retinopathy can be classified as mild, moderate, and severe non-proliferative DR (NPDR) and proliferative DR (PDR). At any stage due to increased vascular permeability and accumulation of intra- and extracellular fluid within the macula, diabetic macular oedema (DME) may appear (*2*). The overall prevalence of any form of DR in the period between 2015 and 2019 was estimated at 27% consisting of 25.2% having NPDR, 1.4% with PDR, and 4.6% with DME (*1*). After 20 years of diabetes duration, almost all patients with type 1 and more than 60% of patients with type 2 diabetes developed some form of retinopathy (*3*). Major risk factors for DR development are hyperglycaemia, hypertension, dyslipidaemia, obesity, sleep apnoea, pregnancy, DM duration, smoking, and genetic factors (*4*-*10*). Various biochemical pathways have been involved in the pathogenesis of DR and despite intensive research; it is still not fully understood (*9*). According to current research, DR is considered as a neurodegenerative, inflammatory, and microvascular complication of diabetes (*9*, *10*). The contribution of inflammatory processes and angiogenesis in structural and molecular alterations associated with DR is gaining increasing attention and has become the focus of many investigations (*10*-*15*).

The primary goal of DR treatment including laser photocoagulation, intravitreal pharmacologic agents, and vitreous surgery is to improve or protect vision (*7*). The most preferred method is intraocular pharmacotherapy, particularly the application of vascular endothelial growth factor (VEGF) inhibitors (*5*, *10*, *12*). Existing methods of DR treatment are predominantly applied at advanced stages of the disease and may be associated with serious side effects. In the early stages, the only available therapeutic approach is strict control of modifiable DR risk factors particularly glycaemic, blood pressure, and serum lipid concentration regulation. Therefore, new diagnostic methods are necessary in order to identify the initial stages of this diabetic complication and monitoring the effects of applied therapy (*5*, *11*, *14*, *15*). Circulating biomarkers could be a valuable tool in detecting early stages of DR, identifying patients most susceptible to its progression and monitoring responses to applied treatment (*4*, *11*, *14*-*17*).

Extensive research has verified the role of inflammation and angiogenesis in the pathogenesis of DR (*4*-*6*, *9*-*15*, *18*). Changes in the concentrations of various pro-inflammatory and angiogenic mediators have been found in serum, aqueous humour (AH), vitreous, retina, and in the tears of patients with DR. Their concentrations are often correlated with the degree of DR implicating that they can be used as biomarkers (*9*, *10*, *12*-*14*, *19*-*39*). Given that a number of these molecules are involved in the formation and development of DR, in this review, we have included potential systemic and local biomarkers which were evaluated based on their role in DR pathogenesis.

## Inflammation and diabetic retinopathy

Inflammation has an important role in the pathogenesis of DR and chronic low-grade inflammation is present in different stages of DR. It is responsible for retinal vasculature damage and takes part in the development of both major causes of visual impairment in diabetes: increased retinal vascular permeability (DME) and neovascularisation (PDR) (*4*-*6*, *9*-*15*, *18*).

Diabetes causes increased local and systemic production of numerous inflammatory molecules involved in DR development such as vascular adhesion molecules, cytokines, chemokines, transcription, and growth factors (*5*, *9*-*15*, *18*-*42*). Elevated concentrations of these molecules induce activation and migration of leukocytes and leukostasis (*4*, *40*, *41*) with subsequent capillary occlusion, retinal hypoxia, and endothelial cell damage. The result of these activities is the blood-retinal barrier (BRB) breakdown which leads to retinal oedema, haemorrhages, exudates, and microaneurysm formation (*4*, *10*, *12*, *14*, *15*, *18*). Activated pericytes are also involved in the retinal inflammatory processes of DR development. They excrete pro-inflammatory mediators, which direct and accumulate immune cells towards the site of retinal inflammation (*42*). There is growing evidence that retinal neurodegeneration may be a relevant pathophysiological mechanism of DR development (*10*, *31*, *43*). Retinal glial cells including astrocytes, Müller cells, and microglia are responsible for providing structural support and maintaining homeostasis in the retina (*43*). Their dysfunction is involved in the early retinal inflammatory process in DR (*12*). Hyperglycaemic stress causes activation of microglial cells (*43*). Retinal neurodegeneration and neuronal apoptosis developed in the early stages of DR causes thinning of the nerve fibre layer and consequent visual impairment and visual loss (*15*).

## Inflammatory and angiogenic interaction in diabetic retinopathy

Angiogenesis is a well-controlled process regulated by balancing proangiogenic (VEGF) and antiangiogenic endogenous factors (*23*, *44*). During this process endothelial cell migration and proliferation with blood vessel maturation and remodelling as well as degradation of extracellular matrix takes place. Degradation of the extracellular matrix is a very important part of this activity since it regulates the entire process (*15*, *18*, *22*, *23*, *45*).

A proangiogenic imbalance causes abnormal growth of new fragile and leaky blood vessels namely neovascularisation (NV) (*18*). One of the major causes of retinal NV is ischemia however, inflammatory cells may also be involved in angiogenic processes by producing angiogenic cytokines and growth factors (*18*, *41*, *44*). Microvascular endothelium activated by cytokines and angiogenic growth factors can express proinflammatory molecules involved in leukocyte mobilisation and activation (*10*, *18*, *44*-*46*). Neovascularization and inflammation share several common mediators and signalling pathways (*18*, *44*). Several chemokines may act as leukocyte attractants and as angiogenic inducers affecting endothelial cells (*18*, *22*, *23*, *44*-*46*). Thus, angiogenesis and inflammation show an interconnection and it can be assumed that inflammation is involved in the early as well as in the advanced stages of DR characterised by increased permeability and NV (*18*, *44*).

## The role of biomarkers in diabetic retinopathy management

Biochemical biomarkers are molecules found in blood or other biological fluid and tissue signifying the existence of an abnormal condition or a disease however may also be indicators of normal biological processes. They may aid in identifying individuals who are at higher risk for developing the disease, patients with early forms of the disease, those with a tendency for disease progression as well as in monitoring the effect of treatment (*16*, *17*, *47*-*49*).

Biomarkers for DR in systemic circulation or local tissues can be an indicator of pathological processes connected with DR based on their role in its development. They can be measured in the blood, vitreous, retina, AH, and recently in tears (Table 1) (*16*, *17*, *19*-*39*, *43*-*56*). The advantage of determining biomarkers in the blood is the availability and larger sample volume of the specimen, routine collection procedures, and standardized analytical techniques as well as the possibility of repetitive analysis. However, as the retina represents a small part of total body mass, a circulating biomarker for DR must be very specific to be relevant. Local biomarkers present the more accurate indicators of the retinal pathology. However, they are only available during surgical procedures, and obtaining a sample of AH or vitreous is an invasive procedure with possible serious complications influencing visual function. An additional problem with the local specimen is the small sample size and the lack of standardized analytical techniques and validation of data (*17*, *21*-*23*, *46*, *50*, *51*). Some clinical and biochemical biomarkers such glycaemia, HbA1c, blood pressure, blood lipid concentrations, and ocular coherence tomography (OCT) are routinely used as biomarkers in the monitoring of patients with diabetes. Among pro-inflammatory and angiogenic factors, we can single out VEGF which has enabled the development of anti-VEGF treatment (*14*, *17*, *26*, *36*, *37*, *39*, *54*). Many other molecules are used in basic science studies investigating the pathogenesis of DR or as surrogate end-points in preclinical studies that explore new therapy options (*6*, *47*, *48*).

**Table 1:** Potential systemic and ocular biomarkers for diabetic retinopathy

**Biomarkers**	**Systemic circulation (references)**	**Ocular fluids**		
		**Tears (references)**	**Aqueous humour (references)**	**Vitreous fluid** **(references)**
CRP	+ (50,51,60,78)	-	-	+ (31)
TNF-α	+ (50,51,69,81)	+ (30,53)	+ (27,32,34)	+ (36,51,59,67)
IL-1β	+ (65)	+ (29)	+ (27,28,32,34)	+ (36,59,62)
IL-6	+ (24,50,69)	+ (29)	+ (27,28,32,34)	+ (22,31,36,59,63,67)
IL-8	+ (24,69)	+ (29)	+ (27,28,32)	+ (22,31,33,36,59)
IL-12	+ 21,50,81)	-	+ (21,27,28)	+ (59)
VEGF	+ (24,69)	+ (29,54)	+ (27,28,34)	+ (22,23,31,54,59,63,91)
PEDF	-	-	+ (92)	+ (63,84,91)
PGF	-	-	+ (27)	+ (39)
ICAM-1	+ (24,69)	-	+ (68)	+ (63,66,67)
VCAM-1	+ (24,69)	-	+ (68)	+ (67,70)
E-selectin	+ (69)	-	-	+ (67)
IGF-1	-	-	+ (94)	+ (93)
TGF-β	+ (94)	-	+ (85)	+ (94)
bFGF	-	+ (29)	+(28)	+ (59,95)
HGF	+ (98,99)	-	+ (27,99)	+ (98)
CTGF	-	-	-	+ (100)
RBP4	+ (78,35)	-	-	-
CXCL10	-	*-*	+ (27,28)	+ (59)
MCP-1	*+* (23,24,69)	+ (29)	*+* (27,28)	+ (22,23,59,63,66)
CCL5	+ (23,24,69)	+ (29)	+ (27,28)	+ (23,59)
CRP – C-reactive protein. TNF-α – Tumour necrosis factor- alpha. IL-1β – Interleukin-1-beta. IL-6 – Interleukin-6. IL-8 – Interleukin-8. IL-12 – Interleukin-12. VEGF –Vascular endothelial growth factor. PEDF – Pigment epithelium-derived factor. PGF – Placental growth factor. ICAM-1 – Intracellular adhesion molecule-1. VCAM-1 –Vascular cell adhesion molecule-1. IGF-1 – Insulin-like growth factor-1. TGF-β –Transforming growth factor beta. bFGF – Basic fibroblast growth factor. HGF –Hepatocyte growth factor. CTGF – Connective tissue growth factor. RBP4 – Retinol-binding protein 4. CXCL10 – chemokine-10. MCP-1 – Monocyte chemotactic protein-1. CCL5 – Chemokine ligand 5.				

Since the existing treatment methods of DR are generally applied in advanced stages of the disease, reliable biomarkers for early detection are needed to enable timely treatment. Future research should be directed at exploring novel DR biomarkers, which should be accessible, non-invasive, economical, and accurate in order to evaluate the presence and progression of DR (*11*, *15*, *17*).

## Collection and analysis of ocular specimens

Evaluating biomarkers in the systemic circulation (Table 2) is a routine procedure at every stage, while the same process with ocular samples represents a challenge. The key problem related to ocular biomarkers is gathering a sufficient volume of ocular specimens such as tears, AH, and vitreous for evaluation (Table 3). Thus, it is essential to optimize collecting, storage, processing, and analysis of obtained samples in order to maximize their use (*47*, *48*). The most readily obtainable ocular specimens are tears and conjunctiva, which usually provide information related to the anterior segment pathology. There are studies in which DR biomarkers have been determined in tears, but there are currently no such studies for the conjunctiva (*29*, *30*, *54*). Diabetic retinopathy biomarkers which more accurately reflect the pathology of the posterior segment of the eye, are those determined from the AH and vitreous (*47*, *48*). However, these are difficult to obtain and entail invasive procedures to collect the sample (*21*-*23*, *26*-*28*, *31*-*34*, *36*-*39*, *47*, *48*, *51*-*54*, *56*, *57*).

**Table 2:** Potential systemic biomarkers for diabetic retinopathy

**Biomarker (analytical technique)**	**Serum concentration (pg/mL)**	**Reference**
CRP (immunoturbidimetric assay)	5.4 ± 5.8	(*19*)
TNF-α (ELISA)	1.7 ± 1.4	(*50*)
IL-1β (ELISA)	0.44 ± 0.13	(*65*)
IL-6 (ELISA)	3.9 ± 1.2	(*50*)
IL-8 (Miliplex X-MAP)	3.0 ± 4.6	(*69*)
IL-12 (Miliplex X-MAP)	1.8 ± 1.6	(*50*)
VEGF (Miliplex X-MAP)	48.76 ± 76.87	(*69*)
ICAM-1 (Miliplex X-MAP)	156.83 ± 89.49	(*69*)
VCAM-1 (Miliplex X-MAP)	900.41 ± 374.81	(*69*)
E-selectin (Miliplex X-MAP)	36.12 ± 37.62	(*69*)
MCP-1 (Miliplex X-MAP)	193.69 ± 133.01	(*69*)
CCL5 (Miliplex X-MAP)	75315.97 ± 63941.70	(*69*)
CRP – C-reactive protein. TNF- α – Tumour necrosis factor- alpha. IL-1β – Interleukin-1- beta. IL-6 – Interleukin-6. IL-8 – Interleukin-8. IL-12 – Interleukin-12. VEGF – Vascular endothelial growth factor. ICAM-1 – Intracellular adhesion molecule-1. VCAM-1 – Vascular cell adhesion molecule-1. MCP-1 – monocyte chemotactic protein-1. CCL5 – Chemokine ligand 5. ELISA - Enzyme-linked immunosorbent assay. Miliplex X-MAP - Multiplex system bead analysis.		

**Table 3:** Potential ocular biomarkers for diabetic retinopathy

**Biomarker**	**Tears** **(reference)**	**Aqueous humour** **(reference)**	**Vitreous fluid** **(reference)**
CRP (pg/mL)	-	-	6.0 ± 2.3^†^ (31)
TNF-α (pg/mL)	2.21 ± 0.04* (*30*)	4.04 ± 1.83^§^ (27)	48.33 ± 4.69^†^ (59)
IL-1β (pg/mL)	16.7 ± 3.2^†^ (29)	1.07 ± 1.03^§^ (27)	1.54 ± 0.14^†^ (59)
IL-6 (pg/mL)	63.3 ± 12.3^†^ (29)	40.64 ± 16.52^§^ (27)	55.20 ± 21.72^†^ (59)
IL-8 (pg/mL)	87 ± 26^†^ (29)	42.20 ± 33.03^§^ (27)	121.84 ± 99.26^†^ (59)
IL-12 (pg/mL)	-	12.85 ± 7.12* (*21*)	37.26 ± 15.92^†^ (59)
VEGF (pg/mL)	149.5 ± 10.4^‡^ (54)	357.02 ± 84.25^§^ (27)	163.31 ± 63.65^†^ (59)
PEDF (pg/mL)	-	1.74 ± 3.68^‖^ (92)	9.4 ± 1.2* (*91*)
MCP-1 (pg/mL)	92.2 ± 10.4^†^ (29)	385.57 ± 147.04^§^ (27)	385.57 ± 147.04^†^ (59)
CCL5 (pg/mL)	35.4 ± 4.6^†^ (29)	1.11 ± 0.35^§^ (27)	3302.78 ± 1238.95^†^ (59)
CRP - C – reactive protein. TNF-α – Tumour necrosis factor- alpha. IL-1β – Interleukin-1-beta. IL-6 – Interleukin-6. IL-8 – Interleukin-8. IL-12 – Interleukin-12. VEGF – Vascular endothelial growth factor. PEDF – Pigment epithelium-derived factor. MCP-1 – monocyte chemotactic protein-1. CCL5 – Chemokine ligand 5. *Enzyme linked-immunosorbent assay. ^†^Bead-based multiplex immunoassay (Bio-Plex Human Cytokine 27-plex panel). ^‡^Quantitative sandwich enzyme immunoassay. ^§^ProcartaPlex immunoassay. ^‖^Western blot analysis.			

Tears may be collected by several non-invasive methods using absorbent materials such as Schirmer strips, minisponges, fire-polished microcapillary tubes, and eyewash. Variations in the results observed between studies can be influenced by the differences in sample collection and storage methods (*47*). During tear sampling, it is essential to avoid activation of the corneal nerves and reflex tearing since it can alter the composition of tear fluid. External factors including the use of topical anaesthesia, artificial tears, contact lens wear, and collection techniques in general may influence tear composition and thus affect the interpretation of the results (*47*, *57*).

Whilst tears are collected by non-invasive procedures, AH samples are gathered invasively *via* aqueous taps during cataract surgery or trabeculectomy. Collection volumes range from 0.1-0.25 mL and in most cases may be sufficient for only one molecule test and a false negative result may occur (*58*). Contact with other eye structures during the collection process may influence the results by contaminating samples with non-AH proteins (*47*).

In order to obtain a larger sample of intraocular fluid, vitreous sampling which usually contains volumes of 1-2 mL is required. This sample is also taken invasively *via* vitreous taps during vitrectomy, by needle aspiration or collection of vitreous refluxes using Schirmer strips, microsponges, and millipore filters after intravitreal injections (*47*, *59*).

Due to the low volume of ocular sample and limitations of the sampling process itself, it is important to set guidelines for the standardization of collection methods, its processing, and storage to enable comparison across studies and increase the reliability of the collected data. Studies undertaken to assess sample integrity and reproducibility have shown that the duration of sample storage should be limited between several days and several weeks (*47*). New protein and gene-based technologies improve sensitivity and enable evaluation of multiple biomarkers in small volumes of tear, AH, or vitreous samples. Improvement of analytical methods opens new opportunities for the use of a single sample for multiple biomarker assessments giving consistent and reliable results (*47*, *59*).

## Inflammatory biomarkers of diabetic retinopathy

Given that inflammation has a significant role in the pathogenesis of DR inflammatory biomarkers have been intensively investigated (*10*, *17*, *22*, *24*, *44*-*46*, *49*-*52*). Various pro-inflammatory factors, such as cytokines and chemokines have been identified at increased concentrations in ocular fluid and tissue namely retina, vitreous, AH, and tears as well as systemic circulation that may be useful as potential biomarkers of DR (*19*-*39*, *52*-*54*, *59*-*77*).

### C-reactive protein

C-reactive protein (CRP) is an acute-phase protein as well as a marker of inflammation and tissue damage. It is produced in the liver and adipose tissue in response to interleukin 6 (IL-6), IL-1β, and tumour necrosis factor-alpha (TNF-α) activity (*9*, *14*). The role of CRP in the pathogenesis of DR has been extensively investigated however clinical studies related to the association between CRP serum and plasma concentrations and DR have obtained diverse results. Some studies confirmed that CRP concentrations are associated with DR in both DM types, whilst others gave opposing results (*13*, *49*-*51*, *60*, *78*). Several studies showed higher concentrations of CRP in patients with PDR in comparison to the patients with NPDR and those findings may support the results of the meta-analysis by Song *et al*. indicating that the CRP concentration may be used as a biomarker of DR severity (*13*, *19*, *49*-*51*, *60*, *78*). However, results regarding the value of CRP as a biomarker for DR due to inconclusive results should be clarified. In a cross-sectional study including 24 patients with PDR conducted by Mallmann *et al.* (*31*) increased vitreous concentrations of CRP were found compared to the control group of 31 non-diabetic patients. The vitreous concentration of CRP was 6.0 ± 2.3 pg/mL which is similar to concentrations found in serum reported by Tomić *et al.* (5.4 ± 5.8 pg/mL) and Zorena *et al.* (2.3 ± 1.0 pg/mL) (*19*, *50*).

### Tumour necrosis factor-α

Tumour necrosis factor-α is a proinflammatory cytokine that increases leukocyte adhesion to retinal endothelium and leukostasis, the production of reactive oxygen species (ROS), permeability of retinal endothelial cells and is implicated in BRB (*4*, *46*, *61*). Increased concentrations of TNF-α have been involved in the development of several chronic inflammatory diseases (*9*, *49*). Current studies showed that patients with DM have higher serum concentrations of TNF-α than healthy controls and these concentrations correlated with the stage of DR with the highest concentrations being found in patients with PDR (*20*, *49*). Additionally, in the vitreous fluid of diabetic patients increased concentrations of TNF-α and the TNF-α expression in epiretinal membranes of patients with PDR were found. This confirms the importance of local TNF-α production for the development of DR (*20*, *46*, *61*, *62*). In a cross-sectional study with 100 participants, Zorena *et al.* found that the risk of NDR in the paediatric population was strongly correlated with serum TNF-α concentration (*50*). In an observational study including 86 patients, Kocabora *et al.* reported increased concentrations of TNF-α in serum, and AH of patients with DME in comparison to healthy controls (*51*). A new approach in the field of retinopathy diagnostics is the assessment of inflammatory mediators in tears (*53*, *54*). Measurement of TNF-α in tears in a prospective study conducted by Costagliola *et al.* showed that TNF-α concentrations were lower in the control group than in diabetic patients with its concentration being correlated to the severity of DR (*53*).

### Interleukin-6

Interleukin-6 is a multifunctional cytokine that regulates inflammation and immune responses, by acting on various cell types including leukocytes, endothelial cells, and fibroblasts (*20*, *25*, *52*, *63*). It is involved in increasing vascular permeability and stimulation of angiogenesis directly and indirectly by inducing expression of VEGF (*9*, *64*). The IL-6 signalling pathway is implicated in the pathogenesis of several inflammatory eye diseases including DR and this association is well documented (*20*, *22*, *23*, *28*, *32*, *52*, *63*, *64*). Vitreous concentrations of IL-6 are associated with DR development and correlates with the severity of retinopathy particularly with PDR and DME (*4*, *9*, *20*, *63*, *65*). Further, several investigations showed a positive correlation between serum concentrations of IL-6 and the presence and stage of DR in both types of DM (*20*, *63*, *65*, *66*, *75*). In a cross-sectional study, including 159 patients with DM Shimizu *et al*. found that serum IL-6 concentrations also significantly related to the severity of ME and could be a predictor of PDR (*75*).

### Interleukin-1 beta

Interleukin-1 beta is a key pro-inflammatory cytokine secreted mostly by monocytes and macrophages which has a substantial role in inflammatory processes involved in DR development (*46*, *64*). Interleukin-1 beta is an unstable molecule and since it is difficult to detect with commercial kits only a few conducted studies exist. The results of these studies showed increased concentrations of IL-1β in serum and ocular fluids of patients with DR and DME (*20*, *27*, *46*, *62*, *65*).

### Interleukin-8

Interleukin-8 or CXCL8 is a pro-inflammatory chemokine, which acts as an activator and stimulator of chemotaxis for neutrophils, monocytes, and lymphocytes and is a powerful promoter of angiogenesis. Increased concentrations of IL-8 have been found in serum as well as vitreous and AH of patients with PDR and DME having a specific role in ME formation associated with diabetes (*20*, *27*, *28*, *33*, *36*, *46*, *64*).

### Cell adhesion molecules

Cell adhesion molecules (CAMs) are involved in leukostasis, in processes of angiogenesis and have a role in the development of vascular complications (*41*, *46*, *76*). They are increased in extraocular and retinal vessels even in the early stages of diabetes and DR, causing increased leukocyte adhesion, vascular leakage, capillary nonperfusion, and endothelial cell damage (*45*, *46*). The most important CAMs are intracellular adhesion molecule-1 (ICAM-1), vascular cell adhesion molecule-1 (VCAM-1), and E-selectin, which are present in high concentrations in the vitreous of patients with PDR (*66*, *67*). The concentrations of ICAM-1 in the vitreous are even higher in patients with active forms of PDR (*66*). The expression of ICAM-1 is increased in the retinal and choroid vessels as well as in the fibrovascular membranes of diabetic patients facilitating the mobilisation of leukocytes (*69*). In a prospective study with 725 African Americans with type 1 DM, Roy *et al.* showed an association between baseline plasma ICAM-1 concentrations with DME and baseline plasma concentration of E-selectin with DR progression (*69*). E-selectin and VCAM-1 are also involved in the pathogenesis of DR and may both act as angiogenic factors on endothelial cells with a direct positive correlation between VCAM-1 and the concentration of VEGF (*18*, *41*, *44*, *67*, *70*). The soluble forms of vascular adhesion molecules were found to be elevated in the vitreous and serum of patients with PDR (*67*, *70*). Their production increased in the presence of high concentrations of glucose and proinflammatory cytokines such as TNF-α and IL-1β (*9*, *46*, *69*). However, some investigations question the direct implication of CAMs in the pathogenesis of DR, and therefore, further research is necessary to clarify their precise role (*6*, *10*, *12*, *14*).

### Retinol-binding protein 4

Retinol-binding protein 4 (RBP4) is an adipokine secreted by hepatocytes and adipose tissue which acts as a specific carrier for vitamin A (*71*). Previous studies have suggested that serum RBP4 concentrations were associated with insulin resistance, metabolic syndrome, impaired glucose tolerance, and type 2 diabetes (*72*-*74*). It has also been associated with inflammatory factors such as CRP and IL-6 (*74*). In an animal model, its overexpression was shown to be connected with early-onset microglia activation, progressive retinal degeneration, and increased expression of pro-IL-18 (*77*). In a cross-sectional study, Li *et al.* found an association of elevated plasma concentrations of RBP4 with DR and vision-threatening DR (VTDR) in 92 Chinese patients with type 2 diabetes, suggesting its possible role in the pathogenesis of DR complications (*78*). Since RBP4 stimulates the expression of proinflammatory molecules in human retinal cells it may be part of the inflammatory pathogenetic process of DR development and may be considered as a biomarker of the early stages of retinopathy (*71*, *77*).

## Angiogenesis related biomarkers

Angiogenesis is the process involving the development of new a vascular network from pre-existing blood vessels. It is characterised with abnormal new vessel formation resulting with hypoxia and vascular leakage (*9*, *15*, *18*, *44*). The neovascularisation of the retina represents a typical feature of PDR. In the process of angiogenesis, numerous mediators are involved with some being considered as potential biomarkers of DR (*18*, *22*, *32*, *33*, *49*, *63*-*65*, *79*-*100*).

### Vascular endothelial growth factor

Vascular endothelial growth factor (VEGF) is a glycoprotein from the family of growth factors, which functions as an effective inducer of retinal vascular permeability and angiogenesis. It is considered as the main angiogenic growth factor implicated in DR development (*79*). The VEGF family consists of six proteins: VEGF-A,-B,-C,-D,-E, and placental growth factor (PGF) with VEGF-A being the best known and most widely used in clinical practice (*80*). VEGF induces retinal ICAM-1 expression and retinal leukocyte adhesion leading to BRB breakdown, capillary non-perfusion, and endothelial cell damage. The imbalance between VEGF and angiogenic inhibitor activity leads to aberrant angiogenesis and proliferation in the retina of patients with DM. Current research results show a significant correlation of VEGF in serum and vitreous with DR and DME (*22*, *24*, *26*, *37*, *54*, *63*, *65*, *82*-*86*). Vascular endothelial growth factor and its receptor were found on the epiretinal membrane in the eyes of patients with diabetes and a correlation between VEGF concentrations and retinopathy activity was established (*37*, *82*). A meta-analysis conducted by Zhou *et al.* showed that VEGF concentrations in the serum correlate to the presence and severity of retinopathy in diabetic patients with the conclusion that serum VEGF may be a potential biomarker for assessing the development and progression of DR (*84*). However, when interpreting the findings, it must be taken into account that serum VEGF concentrations may be influenced by platelet activation (*84*). The assessment of VEGF in tears represents an advancement in screening and diagnosis of DR (*29*, *30*, *51*). Ang *et al.* in a comparative cross-sectional study evaluated the concentrations of VEGF in tears in 88 type 2 DM patients (*54*). Tear samples were collected using Schirmer strips and measured by enzyme-linked immunosorbent assay. Mean tear VEGF concentrations were significantly higher in the NPDR and PDR groups (114.9 ± 8.6 pg/mL and 149.5 ± 10.4 pg/mL, respectively) as compared to the non-DR group (41.2 ± 11.3 pg/mL, P < 0.001) suggesting a significant association with the severity of DR.

### Placental growth factor

Placental growth factor (PGF) as a member of the VEGF family is implicated in pathological angiogenesis, particularly retinal disorders. It can increase the activity of low concentrations of VEGF and indirectly stimulate endothelial cell proliferation and migration (*9*, *83*). Increased concentrations of PGF are observed in the vitreous of patients with PDR with these concentrations being significantly correlated with the concentration of VEGF (*38*, *39*, *79*). Animal models show that PGF mediates both permeability and NV as well as inflammation suggesting that PGF plays an important role in aberrant angiogenesis (*86*). Research studies including patients with the diabetic retinal disease showed that treatment inhibiting both VEGF and PGF provide superior outcomes compared with treatment inhibiting only VEGF (*88*).

### Pigment epithelium-derived factor

Pigment epithelium-derived factor (PEDF) is a glycoprotein with neuroprotective, neurotrophic, and antiangiogenic as well as anti-inflammatory and anti-oxidant activities (*89*, *94*). It may inhibit angiogenesis directly diminishing the expression of the VEGF gene and indirectly by elevating gamma-secretase complex activity effects on VEGF-receptor 1 (VEGFR-1) (*94*). Pigment epithelium-derived factor inhibits the production of ROS, monocyte chemotactic protein-1 (MCP-1), and neutralizes damaging effects of the glycation end products. Experimental studies show that PEDF inhibits neoangiogenesis under conditions where blood concentrations of oxygen concentration are normal and stimulates it in hypoxic situations (*89*, *90*). Previous studies demonstrated that the concentration of PEDF was lower in eyes with DR, particularly in those with PDR. In the vitreous of patients with PDR, the concentration of a soluble VEGF-R1 (sVEGF-R1) was significantly higher whilst the concentration of PEDF was lower when compared to patients with diabetes and no signs of retinopathy (*91*). These findings indicated that the decreased concentration of PEDF in the eyes might be involved in the progression of DR and the degree of retinal NV (*91*, *92*).

### Insulin-like growth factor-1

Insulin-like growth factor-1 (IGF-1) is a polypeptide hormone that has a similar structure and function to insulin and is produced and secreted in the liver, fibroblasts, and chondrocytes. It regulates the proliferation and differentiation of several cell types (*93*). It is involved in the regulation, growth, maturation, and functioning of blood vessels and has an important function in the pathogenesis of DR. The mitotic effect of IGF-1 is the main cause of the growth and proliferation of vascular endothelial cells. The IGF-1 participates in the activation of VEGF in human RPE cells and with its receptor (IGF-1R) is involved in the pathogenesis or progression of proliferative vitreoretinal disorders (*14*, *15*). Experimental studies indicate that IGF-1 stimulates the production of VEGF with significantly increased concentrations in the eyes of patients with PDR compared to the control group (*9*, *93*, *94*).

### Transforming growth factor beta

Transforming growth factor beta (TGF-β), a member of the family of transforming factors, has immunoregulatory activities, and enhances angiogenesis and chondrogenesis (*94*). The concentration of TGF-β showed an association with the severity of microvascular complications and the duration of DM. Patients with DR and NV glaucoma had elevated concentrations of TGF-β in serum (*85*, *94*). Research regarding its concentration in the AH showed similar results. Rusnak *et al.* conducted a prospective cohort study of 61 eyes from 56 patients that suggested that the AH concentrations of IL-6, TGF*β*-1, and VEGF correlate with the severity of PDR (*85*). Patients with NV glaucoma that were refractory to treatment showed higher values of these factors than other PDR patients’ implying that the concentrations of IL-6, TGF*β*-1, and VEGF are correlated with the severity of PDR. However, due to a small group of ten patients with NV glaucoma, these particular results should be interpreted with caution.

### Basic fibroblast growth factor

Basic fibroblast growth factor (bFGF) is a growth factor with mitogen and antigenic activity involved in the survival and maturation of neurons and glial cells as well as tissue repair (*86*). Glial cell line-derived neurotrophic factor stimulates Muller cells to produce bFGF, which in turn promotes endothelial cell proliferation and VEGF production (*9*, *95*). Basic fibroblast growth factor receptor is expressed in the retina and is known to be involved in the formation of epiretinal membranes and in the pathogenesis of PDR (*95*).

### Hepatocyte growth factor

Hepatocyte growth factor (HGF) and its receptor modulate the motility, growth, and morphogenesis of various cell types and have angiogenic activity by inducing the formation of capillary-like tubules (*97*, *98*). In serum and vitreous of patients with PDR elevated concentrations of HGF were found. Shinoda *et al.* in an observational study reported that HGF in AH obtained from 58 diabetic patients during ocular surgery positively correlated with the stage of DR (*99*). Intravitreal HGF concentrations in PDR patients were significantly higher than concentrations found in the control group. Canton *et al.* in their observational study concluded that the high vitreous concentrations of HGF observed in 17 diabetic patients with PDR are not caused by serum diffusion through the BRB rather suggests that intraocular synthesis may be the main contributing factor (*98*).

### Interleukin-12

Interleukin-12 (IL-12) is a cytokine that stimulates proliferation, activation, and cytotoxicity of lymphocytes T and natural killer (NK) cells. It also stimulates these cells to produce interferon-gamma (INF-γ) and TNF-α. Several studies showed that IL-12 has antiangiogenic effects (*21*, *27*, *28*, *94*). *In vitro* studies indicate that maintaining the equilibrium between pro- and anti-inflammatory factors are crucial to sustaining physiological angiogenesis. Shifting this balance in favour of the proangiogenic mediators induces pathological angiogenesis (*81*, *94*). Zorena *et al.* in a cross-sectional study including 126 patients found that in children with type 1 DM and retinopathy the serum concentration of TNF-α was significantly higher and the concentration of IL-12 was significantly lower than in the group without retinopathy suggesting that increased TNF-α production may be the result of insufficient IL-12 concentrations (*81*). They proposed that the balance between the pro- and antiangiogenic cytokines might be one of the preventing factors in the development of DR and nephropathy in children with diabetes. Further, in an observational study conducted by Gverović-Antunica *et al.* a significantly higher concentration of IL-12 was found in the AH of non-treated DR patients in comparison to diabetic patients treated for retinopathy, patients without retinopathy or healthy controls. Since the serum concentrations of IL-12 did not differ considerably between the study groups the conclusion was that this could be due to its local production and secretion (*21*).

### Connective tissue growth factor

Connective tissue growth factor (CTGF) is a cytokine engaged in the stimulation of proliferation, angiogenesis, migration, extracellular matrix production, cell attachment, survival, and apoptosis. It represents a key cell factor that promotes fibrosis with several investigations showing that it is closely related to DR fibrosis development. CTGF promotes the formation of proliferative membranes in PDR and indirectly modulates VEGF expression. Higher concentrations of CTGF and VEGF were found in the vitreous of PDR patients and in the PDR stage, the CTGF and VEGF ratio is a strong predictor of vascular fibrosis transformation (*6*, *9*, *100*).

## Conclusion

Inflammation and angiogenesis play a significant role in the pathogenesis of DR. Serum and ocular biomarkers, which enable the assessment of the presence of inflammatory and angiogenic processes represent useful tools for monitoring the appearance and progression of DR. In the pathogenesis of DME and PDR, VEGF plays an important role with anti-VEGF treatment being an established method of DR therapy. Some patients respond unsatisfactorily to anti-VEGF treatment with the assumption that these patients although having PDR, have undetectable concentrations of VEGF in the vitreous fluid (*55*). This further indicates that VEGF-independent pathways could play a primary role in DR pathogenesis in these particular patients and the improvement of therapeutic methods for blocking other pro-inflammatory and proangiogenic factors could be effective. The identification of reliable and accessible biomarkers for DR would provide valuable information that could be used for the development of new therapeutic strategies applying an individual approach. In this regard, circulating or ocular biomarkers could be particularly useful in detecting patients who would benefit from the existing therapy. Based on this, new methods of treatment could be developed and directed towards personalized medicine.

Limitations in searching for biomarkers of DR could be the fact that their plasma concentrations may reflect the systemic effects of diabetes rather than specific damage in the retina. As such, the focus should shift to assessing circulating concentrations of those expressed predominately in the eye. Ocular fluids particularly aqueous and vitreous humour more reliably reflect the pathophysiological mechanisms of DR development however, obtaining these samples may be associated with serious complications (*21*-*23*, *46*, *51*, *52*). Since tear analysis is readily available, it becomes a promising tool for biomarker identification. Several studies confirm the correlation between the concentration of some biomarkers particularly TNF-α and VEGF in tears and the degree of DR (*53*, *54*). Detection of these biomarkers in tears could be a good non-invasive test for early diagnosis and a predictor of the severity of DR. To date pro-inflammatory and angiogenic molecules are used in basic science studies and it is expected that on the basis of extensive research at least some of these biomarkers in the future will be used in routine clinical practice (*47*, *48*). Given the fact that various pro-inflammatory and proangiogenic factors particularly those that we have evaluated have been implicated in the pathophysiology of DR this opens up a novel area for future investigation of DR biomarker detection.
